# Complete Genome Sequence of a New Halophilic Archaeon, *Haloarcula taiwanensis*, Isolated from a Solar Saltern in Southern Taiwan

**DOI:** 10.1128/genomeA.01529-17

**Published:** 2018-02-01

**Authors:** Pei-Chun Chen, Ting-Wen Chen, Yi-An Han, Wailap Victor Ng, Chii-Shen Yang

**Affiliations:** a Department of Biochemical Science and Technology, National Taiwan University, Taipei, Taiwan; b Molecular Medicine Research Center, Chang Gung University, Guishan, Taiwan; c Bioinformatics Center, Chang Gung University, Guishan, Taiwan; d Department of Biotechnology and Laboratory Science in Medicine, National Yang Ming University, Taipei, Taiwan; e Institute of Biomedical Informatics, National Yang Ming University, Taipei, Taiwan; f Center for Systems and Synthetic Biology, National Yang Ming University, Taipei, Taiwan; g Department of Biochemistry, Kaohsiung Medical University, Kaohsiung, Taiwan

## Abstract

We report here the completion of the genome sequence of a new species of haloarchaea, *Haloarcula taiwanensis*, isolated in southern Taiwan. The 3,721,706-bp genome consisted of chromosome I (2,966,258 bp, 63.6% GC content), chromosome II (525,233 bp, 59.6% GC content), plasmid pNYT1 (129,893 bp, 55.3% GC content), and plasmid pNYT2 (100,322 bp, 55.7% GC content).

## GENOME ANNOUNCEMENT

Haloarchaea (halophilic archaea) are prokaryotes that thrive in high-salt aqueous environments, such as solar salterns and salt lakes. They adopt a microbial rhodopsin (M-Rho) system capable of exerting light-driven ion transportation and photosensing to assist in solar energy harvest, and they adjust their physical position with illumination that favors their optimal survival (1, 2). Among the M-Rho proteins, bacteriorhodopsin is a light-driven outward proton pump that can generate a proton gradient for further ATP generation via F1Fo ATP synthase (3, 4), while halorhodopsin functions as a light-driven inward chloride pump (5) to maintain, at least, the osmolarity of cells. Three types of sensory rhodopsins (SR) have been identified, i.e., SRI (6, 7) and SRII (8), which mediate positive and negative phototaxis responses, respectively, and SRM (9), which senses green light, although its function has yet to be elucidated.

Crystallographic studies unveiled the structures of bacteriorhodopsins (10, 11), halorhodopsins (12, 13), and a sensory rhodopsin II (NpSRII) from *Natronomonas pharaonis* (14). Both NpSRII alone and NpSRII-NpHtrII signal transducer structures were resolved, and the signal transduction from photoreceptor to transducer was proposed (15). On the other hand, possibly due to instability under low-salt conditions, no SRI protein structure has been reported previously. To search for stable SRI protein candidates, we sequenced a new *Haloarcula* species, *Haloarcula taiwanensis*, isolated in southern Taiwan, and found that it possessed a four-rhodopsin system, including the SRI.

Whole-genome shotgun sequences were obtained using a PacBio single-molecule real-time (SMRT) sequencer (16) from Genomics BioSci & Tech (Taipei, Taiwan). The shotgun sequences were assembled using the Hierarchical Genome Assembly Process 3 (HGAP 3) software (17). The assembled contigs contained redundant terminal repeated sequences (RTRS), which were detected by Blast2seq analysis of each assembled *Haloarcula taiwanensis* contig sequence against itself. Within the 17,951- to 55,432-bp imperfect RTRS of the four contigs, multiple polymorphic sites, usually single nucleotide insertions/deletions, were detected. To identify the likely correct sequence, each pair of approximately 240-bp sequences with the polymorphic site near the center were searched against archaeal reference protein sequences using the Blastx algorithm, and the one which matched to protein sequence(s) was considered to have the correct sequence (18). Finally, one of the RTRS copies in each contig was removed by splicing the likely correct sequence in the terminal repeats using the EditPad Lite 7 text editor (Just Great Software).

### Accession number(s).

The sequences of chromosome I, chromosome II, pNYT1, and pNYT2 have been deposited in GenBank under the accession numbers CP019154, CP019155, CP019156, and CP019157, respectively.
